# Early Effects of Mycorrhizal Fungal Inoculum and Fertilizer on Morphological and Physiological Variables of Nursery-Grown *Nothofagus alessandrii* Plants

**DOI:** 10.3390/plants12071521

**Published:** 2023-03-31

**Authors:** Antonio M. Cabrera-Ariza, Patricia Silva-Flores, Marta González-Ortega, Manuel Acevedo-Tapia, Eduardo Cartes-Rodríguez, Götz Palfner, Patricio Ramos, Rómulo E. Santelices-Moya

**Affiliations:** 1 Centro de Investigación y Estudios Avanzados del Maule, Universidad Católica del Maule, Avenida San Miguel 3605, Talca 3460000, Chile; 2 Centro de Desarrollo del Secano Interior, Facultad de Ciencias Agrarias y Forestales, Universidad Católica del Maule, Avenida San Miguel 3605, Talca 3460000, Chile; 3 Centro Tecnológico de la Planta Forestal, Instituto Forestal Sede Biobío, Camino a Coronel Km 7.5, San Pedro de la Paz 4130000, Chile; 4 Laboratorio de Micología y Micorriza, Departamento de Botánica, Facultad de Ciencias Naturales y Oceanográficas, Universidad de Concepción, Concepción 4070386, Chile; 5 Instituto de Ciencias Biológicas, Universidad de Talca, Avenida Lircay s/n, Talca 3460000, Chile

**Keywords:** ruil, fungi, photosynthesis

## Abstract

*Nothofagus alessandrii* (ruil) is an endangered relict species, endemic to the Mediterranean area of Chile, and one of the most threatened trees in the country. Its natural distribution area has been greatly reduced by the effect of human activities; the remaining fragments are mostly intervened and highly deteriorated as a habitat and refuge for the associated biodiversity. In order to produce healthy and resistant nursery plants for recovery and restoration of *N. alessandrii* forests, this study evaluates the early effects of mycorrhizal fungal inoculum (MFI) combined with fertilization on the cultivation of seedlings. The experiment was established under a completely randomized design with a factorial arrangement of the mycorrhizal factors (M0 = without mycorrhizal, M1 = *Thelephora* sp. and M2 = *Hebeloma* sp.) and fertilization (F1 = standard fertilization and F2 = intensive fertilization), with three replicates of each combination, for each type of plant (P1 = plants from one season and P2 = plants from two seasons). Each experimental unit corresponded to a group of 20 plants, with 720 plants in the test. The results indicate that application of fertilizer and MFI significantly affects some growth and photosynthesis parameters of ruil plants in one and two seasons. The morphological parameters obtained in the study show shoot height values ranging between 67 and 91 cm for P1 and between 96 and 111 cm for P2; while, for shoot diameter, values ranged between 7.91 and 8.24 mm for P1 and between 10.91 and 11.49 mm for P2. Although formation of fully developed mycorrhizal roots was not observed during the assay period, we conclude that inoculation of mycorrhizal fungi combined with fertilization could be an efficient strategy to produce a quality plant, in addition to maintaining a high photosynthetic capacity and, therefore, a higher percentage of survival in the field.

## 1. Introduction

The highest biodiversity in Chile is found in the zone of seasonally humid Mediterranean climate (clima mediterraneo pluviestacional, [1]), extending between approximately 33° and 39° SL. This region is also particularly rich in endemic species [2]. The deciduous forests dominated by Nothofagaceae, characteristic for the area, are adapted to the long dry season and play a key role in the retention of water, formation of organic soil and carbon cycle, among others. These forests have a vast variety of ecological niches and habitats for flora, fauna, and associated microbiota [3]. *Nothofagus alessandrii* Espinosa (ruil) is an endemic tree species, emblematic in these ecosystems, belonging to the Maulino Deciduous Forest type [4]. Its natural distribution area has been strongly diminished by soil use change, mainly through the agriculture and timber industries. The remaining fragments are mostly disturbed and highly deteriorated as habitat and refuge for the associated biota [5,6,7].

Ruil is considered a relict species and part of the most ancient lineage of Nothofagus [8,9]. Its actual distribution is limited to a narrow and short strip in the Chilean Coast Range of the Maule Region between approximately 35 and 36° SL [10], where it is in a precarious state of conservation. It has been declared by the Chilean State as a species in danger of extinction [11,12], and by the IUCN as critically endangered [13]. In the past, forests were slashed and burned [14]; currently, the remaining populations are extremely fragmented and typically surrounded by exotic timber plantations, mainly Monterrey pine (*Pinus radiata* D. Don) [15,16], which is highly invasive in ruil forests [17]. The current area where *N. alessandrii* is present does not exceed 314 ha in total [10].

Some initiatives and activities have been developed to conserve and restore *N. alessandrii* forests [18,19,20].

The currently available background on the cultivation of *N. alessandrii* plants is limited and shows variable results [18,21,22,23,24]. A method of increasing worldwide importance in nursery plant production is mycorrhization, which has been shown to increase plant growth and improve plant performance when transplanted to the field [25]. For example, *Hebeloma* and *Thelephora* species are known as pioneer colonizers and are often abundant in nurseries and new plantings [26]. *Thelephora terrestris* Ehrh. ex Fr. has been proven to be important in the uptake of phosphate after one year of establishment of the mycorrhiza [27], while the *Hebeloma* species has been shown to be important in the uptake of water, and the protection of plants against soil-borne pathogens and seedling survival [28,29]. However, the effect of mycorrhization on nursery seedlings and the interaction of mycorrhization with fertilization has not yet been studied in *Nothofagus* [30]. In natural environments, the species of the Nothofagaceae family form ectomycorrhiza (EM) [31,32], reaching more than 70% of fine root colonization by EM fungi [33,34] and are considered obligate ectomycorrhizal [35].

Among the multiple benefits that ectomycorrhizal symbiosis provides for naturally growing and cultivated plants, mitigation of water stress is especially relevant in the Mediterranean climate zone where *N. alessandrii* grows [36]. As for most native species of Chile, there is very little information on this subject for ruil.

The first description of ectomycorrhizal structures on ruil was performed by Garrido [37], and a preliminary inventory of fungi associated with *N. alessandrii* forest was recently published by Palfner et al. [38], yielding about 50 mycorrhiza-forming species, most of them endemic in South American *Nothofagus* forest. However, no mycorrhization experiments with *N. alessandrii* seedlings and their native mycobionts have been published so far.

As for the treatment of nursery seedlings with mycorrhizal fungal inoculum (MFI), it should be noted that due to the usually limited time available for the assay before treated seedlings have to be transplanted to the field, the formation of ectomycorrhizal organs may be still incomplete at the end of the treatment and not be backed by the visible presence of their typical functional morphology. This, however, does not necessarily mean that no physiological changes, induced by the applied MFI, occur in the fine root system, generating positive effects in this early stage on seedling growth. In terms of growth parameters that can be enhanced by mycorrhizal symbiosis, several authors point out that plant size is an important feature of its quality, especially the diameter of the sapling; although, the robustness or slenderness of the sapling is no less important [36,39,40,41]. In the establishment of plantations in Mediterranean environments, which is the condition of the species under study, plants considered robust should have a slenderness index of around six [36] and a high ratio of root/shoot biomass. [40], especially under conditions of low moisture. In order to produce larger but non-slender plants, it is favorable to use bigger containers and a larger growing area. In general, the production of rowan plants has been carried out using 100 or 140 mL cavity containers [22], with variable results in terms of slenderness and size.

Looking at fertilization and its effects, *N. alessandrii* plants of different quality have been shown to be produced with slow delivery fertilizers at the doses suggested by their manufacturer [22,23]. In another study, Acevedo et al. [21] found that fertilization with N doses of 200 mg N L^−1^ was sufficient. On the other hand, higher concentrations of N (400 and 600 mg N L^−1^) led to a luxury consumption that did not produce a higher survival rate at the planting site. Fertilization directly affects plant growth, improving rooting ability at transplanting, and increasing resistance to drought stress, low temperatures, and diseases [21,42,43,44,45]. Different studies have shown positive correlations between fertilization, plant size, and survival in the field, suggesting that the nutritional state might largely explain the success of plantation establishment [46,47,48]. The rise of N and C increases root and stem growth, promoting photosynthesis and ensuring plant establishment [49,50,51]. However, other studies [52,53] have found that higher N fertilization in the nursery could produce a morphological imbalance where stem biomass exceeds root biomass. Under hydric stress conditions, such an imbalance could increase transpiration rates, and reduce stem hydric potential and root hydraulic conductivity [54]. In short, fertilization should be an important factor to consider in plant production. Under the current scenario, there is an urgent need to complement the research so far developed with ruil, which should be oriented to knowledge generation and answers that propose new and effective measures to achieve its recovery and conservation. Therefore, the objective of this study is to evaluate the early effects of mycorrhizal inoculum and fertilizer on morphological and physiological variables of *Nothofagus alessandrii* plants of two different seasons, grown in nurseries.

## 2. Results

### 2.1. Morphological Variables

**Figure 1:**
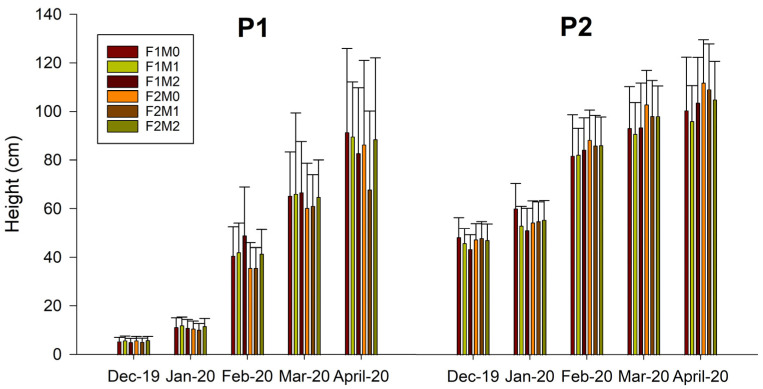
Shoot height of *Nothofagus alessandrii* plants of one and two seasons (P1 and P2) under different combinations of fertilization management (F1 and F2) and application of MFI (M0, M1, and M2).

**Figure 2:**
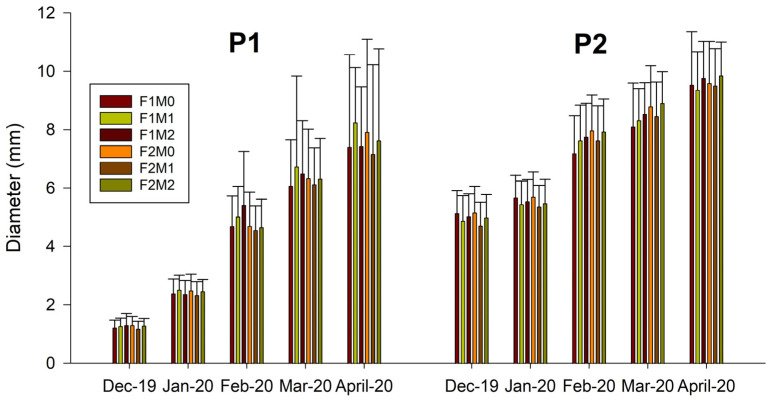
Shoot diameter of *Nothofagus alessandrii* plants of one and two seasons (P1 and P2) under different combinations of fertilization management (F1 and F2) and application of MFI (M0, M1, and M2).

Figure 1 and Figure 2 show shoot growth data (height and diameter, respectively) of nursery plants classified by age class (P1 one season and P2 two seasons), under different combinations of fertilization treatment (F1 and F2), and application of MFI (M0, M1, and M2).

In all treatments, there was sustained growth over time in both shoot height and diameter. A multifactorial ANOVA was performed on all data to verify the effects of the different factors (type of plant, fertilization, and mycorrhization) on growth parameters (Table S1).

**Figure 3:**
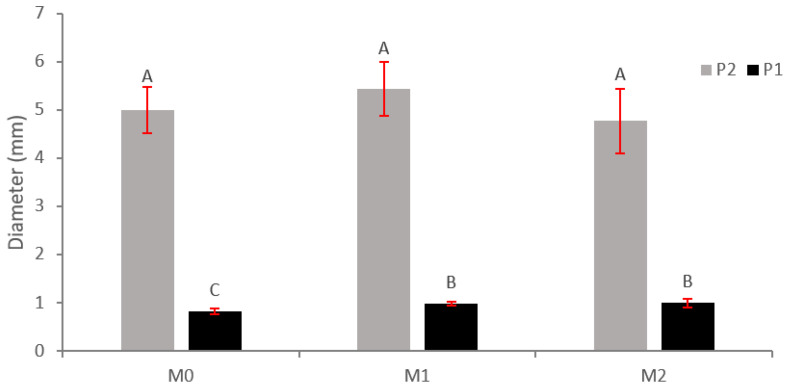
Cumulative increase in shoot diameter (mm) in *N. alessandrii plants* as a function of application of MFI (M0, M1, and M2) and plant type (P1 and P2). Different letters represent significant differences at *p* < 0.05.

For a cumulative increase in height, significant differences were found for plant type, which was evident as they were one- and two-season plants, while, for a cumulative increase in diameter, significant differences were found for plant type, application of MFI, and plant type–MFI interaction (Figure 3). The cumulative increase in height and diameter is greater for P2 plants, possibly due to a greater development of the root system, which makes possible a greater absorption of nutrients and therefore greater growth.

Table 1 shows the results obtained in terms of biomass.

In the case of biomass, after ANOVA analysis, the only significant difference, as expected, was found in the increase in biomass for plants of one and two seasons, the increase being higher in P2 plants. No significant differences were found between the different treatments or the interactions between them (*p* = 0.05).

Regarding the slenderness and stem:root indices, the results are shown in Table 2.

The ANOVA performed (Table S2) indicates significant differences in the MFI–fertilization interaction for the slenderness index in one-season plants and for fertilization in the stem:root index. For two-season plants, there are significant differences in all factors for both indices, except for MFI treatment in the stem:root index.

As for the nutritional analyses, all the data can be found in Table S3, while the ANOVA results can be found in Table S4. As can be seen, MFI treatment had a significant effect only on N. As expected, fertilization had significant differences in most of the nutrients analyzed. In addition, plant type had significant differences in most of the nutrients.

#### Mycorrhizal Analysis

The root system was fully explored in each plant analyzed. None of the plants presented all diagnostic morphological characteristics of ectomycorrhizae. However, the roots looked healthy, active, and with a high presence of root hairs.

**Figure 4:**
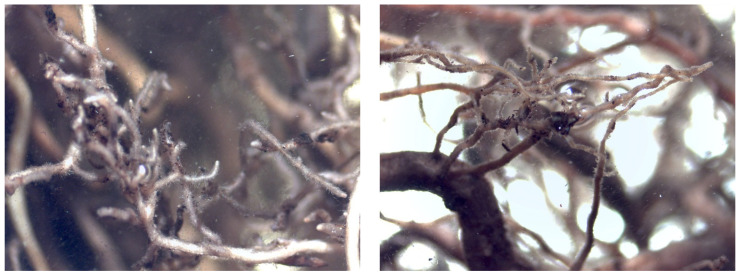
Detail of roots of *Nothofagus alessandrii* seedlings inoculated with *Hebeloma crustuliniforme* (**left**) and *Thelephora terrestris* (**right**).

Figure 4 shows root apices of plants inoculated with *Hebeloma crustuliniforme* (Bull.) Quél. (left) and with *Thelephora terrestris* (right), slender and with the abundant presence of root hairs, but without fungal mantle or periferical mycelium.

**Figure 5:**
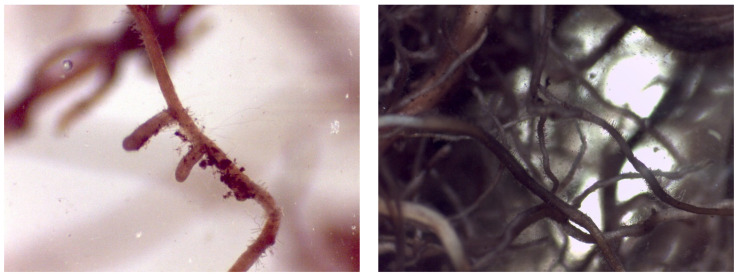
Detail of a short root of a *Nothofagus alessandrii* plant inoculated with *Thelephora terrestris* (**left**) and root of a *Nothofagus alessandrii* plant without inoculation (**right**).

Figure 5 shows lateral fine roots of ruil inoculated with *Thelephora terrestris*, with reduced longitudinal growth, typical for ectomycorrhiza; however, in these roots, the root hairs still persisted, and there was no presence of mantle or Hartig net. This may represent an early stage of mycorrhiza formation.

### 2.2. Physiological Variables

Analyzing the ANOVA performed (Table S5), it is noteworthy that photosynthesis varied as a function of fertilization and the interaction of plant type and MFI treatment; conductance varied as a function of MFI treatment and the interaction between plant type and fertilization; intracellular carbon varied as a function of fertilization and the interaction between plant type and MFI treatment.

**Figure 6:**
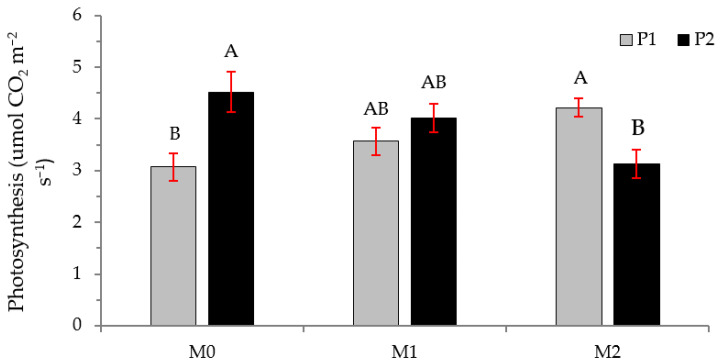
Net photosynthesis for *Nothofagus alessandrii* plants as a function of MFI treatment and plant type. Different letters represent significant differences at *p* < 0.05.

Regarding photosynthesis, Figure 6 shows photosynthesis depending on the type of plant and MFI treatment. Without MFI, P2 plants perform greater photosynthesis; this trend is reversed when the plants are inoculated with *Hebeloma* (M2), and there are no differences in photosynthesis when the plants are inoculated with *Thelephora* (M1).

**Figure 7:**
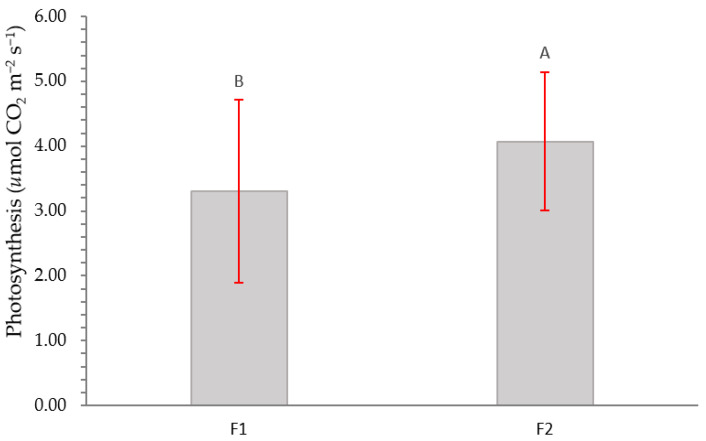
Net photosynthesis for *Nothofagus alessandrii* plants as a function of fertilization type. Different letters represent significant differences at *p* < 0.05.

Figure 7 shows net photosynthesis as a function of fertilization. The values obtained indicate that there is greater photosynthesis in plants with higher fertilization.

**Figure 8:**
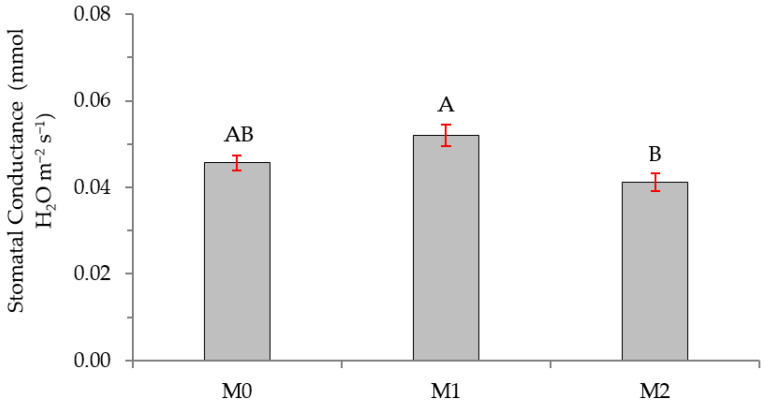
Stomatal conductance in *Nothofagus alessandrii* plants as a function of MFI treatment (M0, M1, and M2). Different letters represent significant differences at *p* < 0.05.

As for stomatal conductance, Figure 8 shows the stomatal conductance as a function of MFI treatment. In this case, there is a greater stomatal conductance in *Thelephora*-inoculated plants vs. *Hebeloma*-inoculated plants. There are no significant differences in the conductance between non-inoculated and inoculated plants.

**Figure 9:**
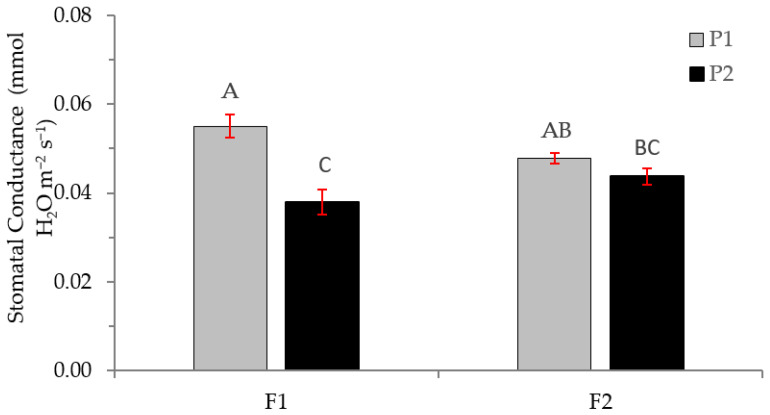
Stomatal conductance in *Nothofagus alessandrii* plants as a function of fertilization (F1 and F2) and plant type (P1 and P2). Different letters represent significant differences at *p* < 0.05.

Figure 9 shows the stomatal conductance as a function of fertilization and the type of plant. The results indicate that the stomatal conductance in plants of one season is higher when a lower fertilization amount is applied. When the amount of fertilizer applied is higher, there are no differences in conductance between plants of one and two seasons.

**Figure 10:**
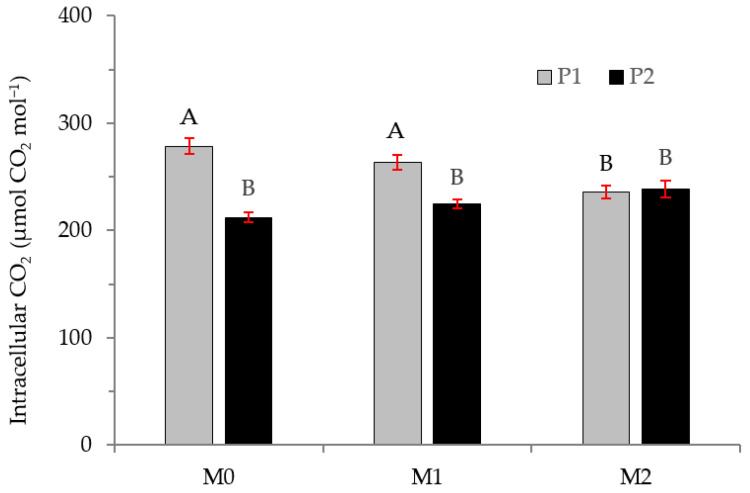
Intracellular CO_2_ in *Nothofagus. alessandrii* plants as a function of MFI treatment (M0, M1, and M2) and plant type (P1 and P2). Different letters represent significant differences at *p* < 0.05.

Finally, for intracellular carbon, Figure 10 shows the results for the interaction between MFI treatment and plant type, with significant differences. Intracellular carbon is higher in plants from one season when they were not inoculated or when they were inoculated with *Thelephora* (M1). However, when plants were inoculated with *Hebeloma* (M2), there were no significant differences in intracellular carbon between plants of one vs. two seasons.

## 3. Discussion

### 3.1. Morphological Variables

The morphological parameters obtained in the study show shoot height values ranging between 67 and 91 cm for one-season plants and between 96 and 111 cm for two-season plants; while, for shoot diameter, values ranged between 7.91 and 8.24 mm for P1 and between 10.91 and 11.49 mm for P2. The heights and diameters obtained are superior to those obtained in other studies. For example, Acevedo et al. [21] examined the effect of different doses of nitrogen fertilization and two container sizes on the cultivation of *N. alessandrii* plants in a nursery. The aforementioned authors obtained heights ranging from 15.4 to 54.3 cm and diameters ranging from 3.84 to 5.51 mm. In another study, Santelices et al. [55] analyzed the effect of cover and fertilization on the initial development of plants during one season. The results show that there was a significant effect of shade on plant development, with better attributes being observed in plants with 35–50% shade, compared to those grown with 80%. However, the height values obtained varied between 25.2 and 31.8 cm, while the diameter value varied between 3.2 and 4 mm. In another work, Santelices et al. [23] studied the response, in terms of germination and growth in a nursery, of viable seeds of *N*. *alessandrii* to different pre-germination treatments, sowing times, and slow-release fertilizers, obtaining heights that varied between 22 and 69 cm and diameters that ranged between 3.1 and 5 mm. Finally, in a study by Santelices et al. [24], they evaluated survival, morphological, and chlorophyll fluorescence differences in *N*. *alessandrii* seedlings grown in a nursery under different shade levels (0%, 18%, 50%, and 80% shade), height values varied between 13.2 and 21.7 cm, and diameters between 2.4 and 3 mm. Although in this case, it must be taken into account that the experiment was developed in 32 weeks.

The growth rate achieved by the plants during cultivation can be considered high. Although there is no official standard for this species, when compared with that of *N*. *nervosa* (Phil.) Dim. et Mil. [56], it is possible to affirm that the plants produced would not only meet the minimum growth demands but would greatly exceed them. For example, the standard contemplates a minimum value of 3 mm for root collar diameter, and this is one of the most important attributes for predicting subsequent plant development in the field [57], particularly under water stress conditions [58]. The high values obtained could be due to the effectiveness of the fertilizer treatment and the interaction with mycorrhizal inoculum. Several studies indicate, for some species, higher growth when a combination of fertilizers and mycorrhization is applied in the nursery [59,60]. In general, the presence of mycorrhizal fungi tends to significantly affect plant growth. However, the direction and magnitude of this effect depend on the particular combination of plant and fungal species and their origin (native or exotic) [61]. In addition, another important factor to take into account in the development of the plants is that they were all produced in bags with a volume of one-liter capacity; the greater root development may have influenced the greater growth in height and diameter of the plants. As for the slenderness index, where low values (i.e., 4–7 for Mediterranean species in Navarro-Cerrillo et al. [39]) tend to reflect a better biomass distribution and better seedling condition [62], it is in an inappropriate range for all levels, implying a higher transpiration rate and water consumption.

In the case of SRI, Navarro-Cerrillo et al. [39] recommended a ratio close to unity to ensure good survival in the early establishment of *Quercus ilex*. According to Villar-Salvador [36], a shoot:root ratio close to one implies a higher probability of seedling survival in drought-prone sites. Plants shift their allocation to shoots if the carbon gain of the shoot is affected by a low level of aboveground resources, such as light and CO_2_. Similarly, plants shift allocation to roots when there is a low level of belowground resources, such as nutrients or water. In this study, seedlings would not be balanced, as they show values above the unit.

#### Application of MFI

Plants that were inoculated with ectomycorrhizal fungi showed some roots with reduced longitudinal growth, in contrast to non-inoculated plants. This modification of plant root architecture in the presence of ectomycorrhizal fungi has been previously described [63,64,65], and is thought to be related to auxin accumulation in the root apex [66,67,68], constituting the first visible step of the mycorrhization process. However, neither Hartig net nor fungal mantle, key diagnostic attributes of a functional ectomycorrhiza, were observed in the examined roots, which may indicate that the duration of the experiment was too short for full formation of the typical EM morphology. The lack of mycorrhization could also be explained as an effect of nitrogen fertilization, indicated by the positive chlorophyll values found in the plants. Several authors argue that high concentrations of nitrogen in the substrate produce a basification of the substrate, which inhibits the formation of mycorrhizae until the substrate becomes acidic [69,70,71,72]. In addition, nitrogen strongly inhibits the growth of extramatrical mycelium [70]. However, *Thelephora terrestris* as well as *Hebeloma crustuliniforme* has been reported to perform well in mycorrhization assays with simultaneous fertilizer application [29,73]. Another explanation could be the irrigation applied to the plants to ensure proper survival and good development for subsequent establishment in the field. In this sense, the substrate being constantly under irrigation can cause the development of “water roots”, which are thicker and less susceptible to mycorrhization [74]. Carrillo [70] found a lower rate of mycorrhization in plants that were growing on a daily-irrigated substrate. In contrast, plants grown with a less intense irrigation regime developed more mycorrhizae due to the greater number of macropores in the substrate. This allows greater aeration and better development of ectomycorrhizae [75]. In this sense, several authors argue that the formation of mycorrhizae is favored by the desiccation of the plants and that these have a better chance of surviving in field conditions after planting [25,70,76,77]. However, as already mentioned, the lack of plant material to propagate plants of the species (i.e., low seed production in the previous season), made it necessary to maintain an irrigation and fertilization regime that ensured the survival of the test. Finally, both EM fungal species used in the assay are sub-cosmopolitan and not specifically associated with *Nothofagus alessandrii*. Although, at least, *T. terrestris* has been reported from other *Nothofagus* species in South America [78], *N. alessandrii* is known to associate with mostly endemic EM fungi [38] and, thus, may have restricted compatibility with *T. terrestris* and *H. crustuliniforme*.

### 3.2. Physiological Variables

Few studies have measured photosynthesis in *N. alessandrii*. Torres-Díaz et al. [79] evaluated the effect of inoculation with a fungal consortium of root endophytes isolated from the Antarctic host plant *Colobanthus quitensis* on ecophysiological performance (photosynthesis, water use efficiency, and growth) in *N. alessandrii* and *Nothofagus glauca* (Phil.) Krasser. These authors found that inoculation with endophytic roots produced significant increases in photosynthetic rates of *N. alessandrii* and *N. glauca*, water use efficiency, and cumulative growth. In another study, in which photosynthesis and stomatal conductance were analyzed in two species of the genus *Nothofagus*, Zúñiga et al. [80], also obtained higher values in photosynthesis, but very similar values in stomatal conductance. Although photosynthesis values for *N. alessandrii* were higher than those obtained in this study, this may be due to the different conditions in the nursery. In the case of this research, the shade provided by the raschel mesh may have decreased the values. In another study, Sebastiana et al. [81] studied whether cork oak yield under drought could be improved by colonization with the ectomycorrhizal fungus *Pisolithus tinctorius* (Pers.) Coker and Couch. In this case, inoculation had no effect on photosynthesis, suggesting that the symbiosis with *P. tinctorius* was not effective in inducing stomata opening to sustain photosynthesis under conditions of low water availability, as has been suggested in other studies [82,83].

Although morphological changes in the root system, which can be an efficient strategy to increase water uptake under drought conditions, were not evaluated, statistical analyses indicated the influence of the applied mycorrhizal inoculum on relative growth and photosynthesis. It has been suggested that endophyte-induced variations in the rhizosphere, such as in the production of sugars, proteins, and/or enzymes that prevent cell damage to membranes, allow some plants to cope with the stressful environmental conditions that can be found in Mediterranean ecosystems [84]. Therefore, inoculation of mycorrhizal fungi in roots could be an efficient strategy to maintain a high photosynthetic capacity and, hence, a higher percentage of survival in the field.

## 4. Materials and Methods

### 4.1. Plant Production

*N. alessandrii* seedlings were produced at the Forest Plant Production Center of the National Forestry Institute INFOR at San Pedro de La Paz, Chile. These were grown in 13 cm × 25 cm polyethylene bags with a volume of approximately 1000 ccs and pine bark compost substrate of G10 granulometry (<10 mm), characterized by a pH of 5.5; organic matter = 56.5%; total nitrogen = 0.6%; carbon–nitrogen ratio = 27; N–NO_3_^−^ = 140 mg kg^−1^; N–NH_4_^+^ = 67.8 mg kg^−1^; 27% of water retention; and 25% of aeration porosity. This substrate was sterilized in three autoclave cycles at 121 °C for 30 min for each cycle.

Two age classes of plants were used, viz.: seedlings of the season (P1) and second-year seedlings (P2); P1 seedlings were sown on 25 September 2019, in previously sterilized sand, with systematic irrigation during the germination period. After having their first pair of true leaves, these plants were transferred to polyethylene bags. P2 seedlings were sown on 18 October 2018. In order to avoid the use of plants with previous, spontaneous mycorrhization in the assays, we monitored selected plants for the visible presence of mycelium in the substrate and on fine roots under a dissecting microscope at the Mycology Laboratory of the Department of Botany, Universidad de Concepción. After this evaluation, the plants were also transferred to bags.

Prior to the experimental treatments, both plant groups (P1 and P2) were systematically irrigated for 1 month in order to allow the formation of new roots inside the containers. For the assays, we used a 50% raschel mesh as protection from direct light, and planting tables and surrounding areas were disinfected using a 1% solution of Captan 80 WP applied with a back pump. In P2 plants, apical pruning was performed at the time of establishing the test, eliminating the new foliage formed under greenhouse conditions. From the start of the assay setup, and by using previously calibrated soil moisture sensors, an irrigation regime was carried out considering 70%, 60%, and 50% of substrate retention capacity, respectively, according to the methodology proposed by Cartes et al. [85].

#### Fertilization Treatments

The application of two contrasting fertilization schedules (F1 and F2) in the application of macro and micronutrients was considered, with F1 being lower than F2 (Table 3). Fertilizer was prepared by using soluble salts (Urea, Sodium nitrate, Ammonium nitrate, Monopotassium phosphate, Calcium nitrate, Magnesium sulfate heptahydrate, Ferrous sulfate pentahydrate, manganese sulfate tetrahydrate, copper sulfate heptahydrate, zinc sulfate heptahydrate, Sodium molybdate Dihydrate, Sodium borate decahydrate), following the methodology proposed by Landis [86]. To apply the fertilizers, fertigation was used throughout the growth period of the plants.

### 4.2. Mycelium Production and Inoculation

Mycelial biomass production of *Hebeloma crustuliniforme* (strain IF: 732005) and *Thelephora terrestris* (strain IF: 711004) for inoculation of *Nothofagus alessandrii* plants was produced in a 5 L (LiFlux Gx) stirred tank bioreactor culture, belonging to the Fungal Biotechnology Laboratory, Universidad de Concepción, Campus Los Ángeles. Both EM species were selected being known as early-stage colonizers with a broad tree host range, having been successfully used under greenhouse conditions with fertilization regimes [29,73], and also having been reported from natural environments in Chile [78]. The initial inoculum was grown for 15 days before transferring the mycelium to the bioreactor. Modified Melin–Norkrans medium (MMN, with glucose reduced to ¼ of the original) was used. The following parameters were monitored: temperature 24 °C, pH 5.5 (adding NaOH 0.5 N in case of acidification of the medium during fungal growth), constant agitation at 100 rpm, airflow 0.5 L min^−1^, and dissolved oxygen (% OD) in excess of 60% [82,87]. The final culture volume was 2.5 L and the initial inoculum consisted of 10% of pre-cultured (*v*/*v*) mycelium (2.25 L plus 250 mL of initial inoculum). Finally, the total mycelial biomass produced was harvested and used for plant inoculation.

Inoculation with *Thelephora* (M1) and *Hebeloma* (M2) was performed in two instances: the first was carried out together with the experimental setup (on 17 December 2019), applying 6 mL inoculum per plant taken from a 10% suspension of the harvested mycelium of each strain; the second inoculation was carried out on 4 February 2020 following the same procedure. For each of the inoculation instances, distilled water was used to suspend the mycelium. In the case of the experiments without inoculation (M0), the same volume of distilled water was applied.

### 4.3. Evaluation of Morphological Variables

The collar diameter (DOC) and stem length (SL) of all plants were measured. These evaluations were repeated monthly until the end of the experiment. Likewise, biomass by components (root, stem, and foliage) was evaluated at the end of the test, with a random sampling of three plants per experimental unit. In the case of root and stem biomass, drying was carried out in a forced ventilation oven at 105 °C until constant weight. For foliage biomass, drying was carried out in a forced ventilation oven at 65 °C until constant weight. Dry weights were recorded to the nearest 0.01 g.

In addition, the slenderness index (SI) and the stem:root index (SRI) [88] were calculated, according to the following Formulas (1) and (2):

SI = SL (cm)/DOC (mm)(1)

SRI =AB (g)/RB (g)(2)

where AB is the aerial biomass and RB is the root biomass.

On the other hand, leaf samples were taken in May 2020 for a complete nutritional analysis. The analyses were carried out at the Soil and Crop Technology Center of the University of Talca, following the established protocols (http://www.ctsyc.cl/ accessed on 17 July 2020). The objective was to verify if mycorrhization has a nutritional effect on the plant.

### 4.4. Evaluation of Physiological Variables

For the analysis of gas exchange during December, a sample of three plants per experimental unit was taken, and the parameters net photosynthesis (An, umol CO_2_ m^−2^ s^−1^), stomatal conductance (gs, mol H_2_O m^−2^ s^−1^), and intracellular CO_2_ (μmol CO_2_ mol^−1^) were measured for each plant. Measurements were taken between 11:00 and 14:00 (local time) with a portable photosynthesis system (LICOR model Li-6400XT), under illumination of 1000 mmol m^2^ s^1^, which was obtained with red/blue light source (LICOR model 6400-02B).

During the measurements, the relative humidity inside the measuring chamber varied between 25 and 26% (vapor pressure deficit of 1.7 to 2.2 kPa). CO_2_ concentration in the chamber was adjusted up to 400 ppm. The block temperature was set at 25 °C, resulting in leaf temperatures between 22 and 24 °C, and the airflow through the chamber was 500 mL min^−1^. Readings were taken once the equilibrium state was reached, usually less than 3 min per plant.

### 4.5. Evaluation of Mycorrhization

For mycorrhization analysis, the entire root system of three plants per experimental unit was harvested, washed with tap water, and stored in 50 mL bottles with 70% ethanol for later analysis. The root systems were subsequently removed from the alcohol, and placed in trays with tap water, in order to stretch the roots and remove the remaining soil. They were then scanned under a Leica EZ4HD stereo microscope for diagnostic attributes of successful mycorrhization, such as reduced longitudinal growth, absence of root hairs, and presence of fungal mantle and/or peripheral mycelium, with characteristic coloration and texture.

### 4.6. Data Analysis

The test was set up under a completely randomized design with a factorial arrangement of the factor mycorrhizal inoculum (M0, M1, and M2) and fertilization (F1 and F2), with three replicates of each combination, for each type of plant (P1 and P2). Each experimental unit corresponded to a group of 20 plants, with a total amount of 720 plants in the test.

The data analysis for the variables of growth and biomass (collar diameter, stem length, and total biomass, foliage, stem, and roots), were carried out based on the cumulative increment at the end of the evaluations and relative to the initial size of the plants (relative cumulative increment). For all the variables (including physiological), the analyses were performed by means of generalized linear models, using PROC GLIMMIX (SAS Institute, Cary, NC, USA), with distribution selection based on the Akaike information criterion. The model structure considered factor Plant type (P1 and P2; two levels), Fertilization (F1 and F2; two levels), Inoculation (M0, M1, M2; three levels), and interactions, with three replicated in each treatment. The identification of outliers was performed from the interquartile range, considering the adjustment of the degrees of freedom using the Satterthwaite approximation. The multiple comparisons of means for significant effects were performed by Tukey’s adjustment with a significance level of 0.05.

## 5. Conclusions

The results indicate that both fertilization and application of MFI have an effect on the growth and photosynthesis of *N. alessandrii* plants in one and two seasons. Although no completely formed mycorrhizal organs could be observed to be formed in the seedlings during the assay, measured effects may be early responses to the combined treatment. Therefore, the inoculation of seedlings with mycorrhizal fungi, combined with a specific fertilization regime, according to our observations, is an efficient strategy in order to produce a quality plant with a high photosynthetic capacity and, therefore, a higher potential of survival in the field.

Nevertheless, it is necessary to continue investigating the mycorrhization of this species and, above all, to analyze the long-term performance in the areas of the natural distribution of the species.

## Figures and Tables

**Table 1:** Biomass and their standard deviations of *Nothofagus alessandrii* seedlings by components (root, stem, and leaves) for one- and two-season plants (P1 and P2) with different fertilization (F1 and F2) and MFI management (M0, M1, and M2).

Treatment	Roots Biomass (gr)	Stems Biomass (gr)	Leaves Biomass (gr)	Total Biomass (gr)
P2F1M0	10.19 ± 3.74	19.65 ± 6.62	7.52 ± 3.28	37.37
P2F1M1	11.71 ± 2.85	21.94 ± 5.03	9.03 ± 2.37	42.68
P2F1M2	11.09 ± 3.98	19.97 ± 4.72	8.28 ± 2.69	39.34
P2F2M0	10.49 ± 2.20	21.86 ± 4.75	8.17 ± 2.87	40.52
P2F2M1	9.53 ± 3.74	18.41 ± 6.83	7.34 ± 3.99	35.28
P2F2M2	10.21 ± 3.73	19.97 ± 6.44	7.78 ± 3.18	37.96
P1F1M0	5.12 ± 2.23	9.50 ± 3.41	6.34 ± 2.17	20.96
P1F1M1	4.45 ± 2.38	8.51 ± 3.92	5.76 ± 2.42	18.72
P1F1M2	4.42 ± 2.44	8.00 ± 3.66	5.54 ± 2.25	17.96
P1F2M0	3.99 ± 2.03	8.26 ± 3.95	5.71 ± 2.54	17.97
P1F2M1	3.42 ± 1.79	7.13 ± 3.42	5.00 ± 2.09	15.55
P1F2M2	3.35 ± 1.85	7.12 ± 3.75	4.88 ± 2.23	15.35

**Table 2:** Slenderness index (SI) and Stem:Root index (SRI) and their standard deviations for plants of one (P1) and two (P2) seasons of *Nothofagus alessandrii* under different treatments of fertilization (F1 and F2) and application of MFI (M0, M1, and M2).

Index	P1F1M0	P1F1M1	P1F1M2	P1F2M0	P1F2M1	P1F2M2
**SI**	12.51 ± 3.32	11.07 ± 2.55	11.13 ± 2.73	11.05 ± 2.99	11.82 ± 2.20	8.50 ± 3.65
**SRI**	3.17 ± 0.68	2.78 ± 0.89	2.81 ± 0.85	3.39 ± 0.36	3.98 ± 0.95	3.91 ± 0.87
	**P2F1M0**	**P2F1M1**	**P2F1M2**	**P2F2M0**	**P2F2M1**	**P2F2M2**
**SI**	9.86 ± 1.92	9.39 ± 1.65	9.49 ± 1.93	9.63 ± 1.62	9.87 ± 1.88	9.46 ± 1.70
**SRI**	2.52 ± 0.58	2.28 ± 0.39	2.99 ± 0.58	3.08 ± 0.68	3.19 ± 0.79	2.77 ± 0.46

**Table 3:** Fertilization schemes applied.

Fertilization Scheme	Total N (mg L^−1^)	N–NO_3_^−^ (mg L^−1^)	N–NH_4_^+^ (mg L^−1^)	P (mg L^−1^)	K (mg L^−1^)	Ca (mg L^−1^)	Mg (mg L^−1^)
**F1**	50	25	25	25	32.4	15	12.5
**F2**	200	100	100	100	127	60	50
**Fertilization Scheme**	**S** **(mg L^−1^)**	**Fe** **(mg L^−1^)**	**Mn** **(mg L^−1^)**	**Cu** **(mg L^−1^)**	**Zn** **(mg L^−1^)**	**Mo** **(mg L^−1^)**	**B** **(mg L^−1^)**
**F1**	17.2	2.5	2.5	0.5	2.5	0.5	0.5
**F2**	79.7	10	10	2	10	2	2

## Data Availability

No new data were created or analyzed in this study. Data sharing is not applicable to this article.

## Supplementary Materials

The following supporting information can be downloaded at: https://www.mdpi.com/article/10.3390/plants12071521/s1, Table S1: Significance level for the main effects (TP = Plant type, F = Fertilization, M = mycorrhization) and their interactions in the morphological attributes height and diameter of *Nothofagus alessandrii*.; Table S2: Significance level for the main effects (TP = Type of plant, F = Fertilization, M = mycorrhization) and their interactions in the morphological attributes Stem:Root Index (SRI) and Slenderness Index (SI) of *Nothofagus alessandrii* seedlings; Table S3: Macro and micro nutrients concentration in leaves of *Nothofagus alessandrii* in one and two-season plants (P1 and P2) (TP = Type of plant, F = Fertilization, M = mycorrhization); Table S4. The significance level for the main effects (TP = Type of plant, F = Fertilization, M = mycorrhization) and their interactions on the concentration of nutrients in the leaves of *Nothofagus alessandrii* in one- and two-season plants (P1 and P2); Table S5. Level of significance for main effects (TP = Plant type, F = Fertilization, M = mycorrhization) and their interactions on photosynthesis, stomatal conductance, and intracellular CO_2_ of *Nothofagus alessandrii* plants.

## References

[1] Luebert F., Pliscoff P. (2017). Sinopsis Bioclimática y Vegetacional de Chile.

[2] Myers N., Mittermeier R.A., Mittermeier C.G., da Fonseca G.A.B., Kent J. (2000). Biodiversity hotspots for conservation priorities. Nature.

[3] Arroyo M.T.K., Riveros M., Peñaloza A., Cavieres L., Faggi A.M., Lawford R.G., Alaback P.B., Fuentes E. (1996). Phytogeographic relationships and regional richness patterns of the cool temperate rainforest flora of southern South America. High-Latitude Rainforests and Associated Ecosystems of the West Coasts of the Americas: Climate, Hydrology, Ecology and Conservation.

[4] Gajardo R. (1983). Sistema Básico de Clasificación de la Vegetación Nativa Chilena.

[5] San Martín J., Sepúlveda C. (2002). Diagnóstico del estado actual de los fragmentos de Nothofagus alessandrii, ruil, Fagaceae (=Nothofagaceae), de la Región del Maule, Chile Central.

[6] Olivares P., San Martín J., Santelices R. (2005). Ruil (Nothofagus alessandrii): Estado del Conocimiento y Desafíos Para su Conservación.

[7] Burgos A., Grez A.A., Bustamante R.O. (2008). Seed production, pre-dispersal seed predation and germination of *Nothofagus glauca* (*Nothofagaceae*) in a temperate fragmented forest in Chile. For. Ecol. Manag..

[8] Serra M.T., Gajardo R., Cabello A. (1986). Programa de Protección y Recuperación de la Flora Nativa de Chile, Ficha Técnica de Especies Amenazadas: Nothofagus Alessandrii Espinosa “Ruil” (Fagaceae), Especie en Peligro.

[9] Knapp M., Stöckler K., Havell D., Delsuc F., Sebastiani F., Lockhart P.J. (2005). Relaxed Molecular Clock Provides Evidence for Long-Distance Dispersal of Nothofagus (Southern Beech). PLoS Biol..

[10] Santelices R., Drake F., Mena C., Ordenes R., Navarro-Cerrillo R.M. (2012). Current and potential distribution areas for *Nothofagus alessandrii*, an endangered tree species from central Chile. Cienc. Investig. Agrar..

[11] Benoit I. (1989). Libro Rojo de la Flora Terrestre de Chile.

[12] Hechenleitner P., Gardner M., Thomas P., Echeverría C., Escobar B., Brownless P., Martínez C. (2005). Plantas Amenazadas del Centro-sur de Chile. Distribución, Conservación y Propagación.

[13] Barstow M., Echeverría C., Baldwin H., Rivers M.C. Nothofagus Alessandrii. The IUCN Red List of Threatened Species 2017: E.T32033A2808995. http://www.iucnredlist.org/details/32033/0.

[14] Donoso C., Landaeta E. (1983). Ruil (*Nothofagus alessandrii*), a threatened Chilean tree species. Environ. Conserv..

[15] Bustamante R.O., Castor C. (1998). The decline of an endangered temperate ecosystem: The ruil (*Nothofagus alessandrii*) forest in central Chile. Biodivers Conserv..

[16] Bustamante R., Grez A. (1995). Consecuencias ecológicas de la fragmentación de los bosques nativos. Ambiente y Desarro..

[17] Bustamante R., Simonetti J. (2005). Is *Pinus radiata* invading the native vegetation in Central Chile? Demographic responses in a fragmented forest. Biol. Invasions.

[18] San Martín J., Santelices R., Henríquez R., Edición S., Donoso C. (2013). Nothofagus alessandrii Espinosa, Ruil. Familia: *Nothofagaceae*. Las Especies Arbóreas de los Bosques Templados de Chile y Argentina: Autoecología.

[19] González M., Becerra-Rodas C., Molina I., Lara A., Little C., San Martín J. (2022). Restauración de bosques de Rui: Línea base, desarrollo e implementación del plan de restauración en predio El Desprecio, Región del Maule. Los Bosques Relictos de Ruíl: Ecología, Biodiversidad, Conservación y Restauración.

[20] Quiroz I., San Martín J. (2022). Técnicas de restauración ecológica con ruil. Los Bosques Relictos de Ruíl: Ecología, Biodiversidad, Conservación y Restauración.

[21] Acevedo M., Alvarez C., Cartes E., Dumroese R.K., Gonzalez M. (2020). Production and establishment techniques for the restoration of *Nothofagus alessandrii*, an endangered keystone species in a Mediterranean forest. New For..

[22] Santelices R., Navarro-Cerrillo R.M., Drake F. (2009). Caracterización del material forestal de reproducción de cinco procedencias de *Nothofagus alessandrii* Espinosa una especie en peligro de extinción. Interciencia.

[23] Santelices R., Navarro-Cerrillo R.M., Drake F. (2011). Propagation and seedling cultivation of the endemic species *Nothofagus alessandrii* Espinosa in Central Chile. Restor. Ecol..

[24] Santelices R., Espinoza S., Cabrera A. (2015). Effect of four levels of shade on survival, morphology and chlorophyll fluorescence of Nothofagus alessandrii container-grown seedlings. Iforest—Biogeosciences For..

[25] Pera J., Alvarez I., Parlade J. (1998). Eficacia del inóculo miceliar de 17 especies de hongos ectomicorrícicos para la micorrización controlada de: *Pinus pinaster, Pinus radiata* y *Pseudotsuga menziessii*, en contenedor. Investig. Agraria. Sist. Y Recur. For..

[26] Palacios Y.M., Palfner G., Hernández C.E. (2012). The ectomycorrhizal community in a chronosequence of *Pinus radiata* (Pinophyta: Pinaceae) of the transitional Mediterranean-temperate climatic zone of central Chile. Rev. Chil. Hist. Nat..

[27] Kammerbauer H., Agerer R., Sandermann H. (1989). Studies on ectomycorrhiza. Trees.

[28] Annesi T., Motta E. (1998). Hebeloma inoculation on Norway spruce seedlings in solarized and infested soil. Eur. J. For. Pathol..

[29] Siemens J.A., Calvo-Polanco M., Zwiazek J.J. (2011). Hebeloma crustuliniforme facilitates ammonium and nitrate assimilation in trembling aspen (*Populus tremuloides*) seedlings. Tree Physiol..

[30] van Galen L.G., Lord J.M., Orlovich D.A., Larcombe M.J. (2021). Restoration of southern hemisphere beech (Nothofagaceae) forests: A meta-analysis. Restor. Ecol..

[31] Godoy R., Palfner G. (1997). Ectomicorrizas en Nothofagus alpina (Poepp. & Endl.) Oerst. y N. dombeyi (Mirb. Oerst.) del sur de Chile. Boletín Micológico.

[32] Palfner G. (2001). Taxonomische Studien an Ektomykorrhizen aus den Nothofagus-Wäldern Mittelsüdchiles.

[33] Diehl P. (2006). Indicadores de Conservación de Nitrógeno y Fósforoen Especies Arbóreas de la Región Andino-Patagónica. Ph.D. Thesis.

[34] Diehl P., Mazzarino M., Fontenla S. (2008). Plant limiting nutrientsin Andean–Patagonian woody species: Effects of interannual rain-fall variation, soil fertility and mycorrhizal infection. Forest Ecol. Manag..

[35] Nouhra E., Palfner G., Kuhar F., Pastor N., Smith M., Pagano M., Lugo M. (2019). Ectomycorrhizal Fungi in South America: Their Diversity in Past, Present and Future Research. Mycorrhizal Fungi in South America.

[36] Villar-Salvador P., Rey-Benayas J.M., Espigares-Pinilla T., Nicolau-Ibarra J.M. (2003). Importancia de la calidad de planta en los proyectos de revegetación. Restauración de Ecosistemas Mediterráneos.

[37] Garrido N., Bibliotheca Mycologica J. (1988). Agaricales s.l. und ihre Mykorrizen in den Nothofagus-Wäldern Mittelchiles.

[38] Palfner G., Casanova A., Salazar V., Riquelme A., Santelices R., San Martín J. (2022). Hongos no liquenizados: Diversidad, funciones, conservación y usos. Los Bosques Relictos de Ruil: Ecología, Biodiversidad, Conservación y Restauración.

[39] Navarro-Cerrillo R., Villar-Salvador P., Del Campo A., Cortina J., Peñuelas J., Puértolas J., Savé R., Vilagrosa A. (2006). Morfología y establecimiento de los plantones. Calidad de Planta Forestal Para la Restauración en Ambientes Mediterráneos, Estado Actual de Conocimientos.

[40] Thomson S., Durvea M.L. (1985). Seedling morphological evaluation—What you can tell by looking. Evaluating Seedling Quality: Principles, Procedures, and Predictive Abilities of Major Tests, Proceedings of the Workshop Held 16–18 October 1984.

[41] Mexal J., Landis T.D., Rose R., Campbell S.J., Landis T.D. (1990). Target Seedling Concepts: Height and Diameter. Target Seedling Symposium, Proceedings of the Western Forest Nursery Association, Roseburg, OR, USA, 13–17 August 1990.

[42] Malik V., Timmer V.R. (1998). Biomass partitioning and nitrogen retranslocation in black spruce seedlings on competitive mixed wood sites: A bioassay study. Can. J. For. Res..

[43] Floistad I.S., Kohmann K. (2004). Influence of nutrient supply on spring frost hardiness and time of bud break in Norway spruce (*Picea abies* (L.) Karst.) seedlings. New For..

[44] Dumroese R.K., Page-Dumroese D.S., Salifu K.F., Jacobs D.F. (2005). Exponential fertilization of Pinus monticola seedlings: Nutrient uptake efficiency, leaching fractions, and early outplanting performance. Can. J. For. Res..

[45] Salifu K.F., Jacobs D.F. (2006). Characterizing fertility targets and multi-element interactions in nursery culture of *Quercus rubra* seedlings. Ann For. Sci..

[46] Puértolas J., Gil L., Pardos J.A. (2003). Effects of nutritional status and seedling size on field performance of Pinus halepensis planted on former arable land in the Mediterranean basin. Forestry Int. J. For. Res..

[47] Villar-Salvador P., Planelles R., Enríquez E., Rubira J.P. (2004). Nursery cultivation regimes, plant functional attributes, and field performance relationships in the Mediterranean oak *Quercus ilex* L.. For. Ecol. Manag..

[48] Oliet J.A., Planelles R., Artero F., Valverde R., Jacobs D.F., Segura M.L. (2009). Field performance of Pinus halepensis planted in Mediterranean arid conditions: Relative influence of seedling morphology and mineral nutrition. New For..

[49] Grossnickle S.C. (2005). Importance of root growth in overcoming planting stress. New For..

[50] del Campo A.D., Navarro R.M., Ceacero C.J. (2010). Seedling quality and field performance of commercial stocklots of containerized holm oak (*Quercus ilex*) in Mediterranean Spain: An approach for establishing a quality standard. New For..

[51] Trubat R., Cortina J., Vilagrosa A. (2011). Nutrient deprivation improves field performance of woody seedlings in a degraded semi-arid shrubland. Ecol. Eng..

[52] Trubat R., Cortina J., Vilagrosa A. (2008). Short-term nitrogen deprivation increases field performance in nursery seedlings of *Mediterranean woody* species. J. Arid. Environ..

[53] Cortina J., Vilagrosa A., Trubat R. (2013). The role of nutrients for improving seedling quality in drylands. New For..

[54] Hernández E.I., Vilagrosa A., Luis V.C., Llorca M., Chirino E., Vallejo V.R. (2009). Root hydraulic conductance, gas exchange and leaf water potential in seedlings of *Pistacia lentiscus* L. and *Quercus suber* L. grown under different fertilization and light regimes. Environ. Exp. Bot..

[55] Santelices R., Navarro-Cerrillo R., Drake F., Mena C. (2011). Efecto de la cobertura y de la fertilización en el desarrollo de plantas de *Nothofagus alessandrii* cultivadas en contenedor. Bosque.

[56] INN (INdN) (2005). NCH2957/0 Madera. Material de Propagación de Uso Forestal. Parte 0: Producción y Comercialización.

[57] Ritchie G., Landis T., Dumroese R., Haase D., Landis T.D., Haase D.L. (2010). Chapter 2: Assessing plant quality. The Container Tree Nursery Manual, Volume 7. Seedling Processing, Storage, and Outplanting.

[58] South D.B., Rakestraw J.L., Lowerts G.A. (2001). Early gains from planting large-diameter seedlings and intensive management are additive for loblolly pine. New For..

[59] Salto C.S., Sagadin M.B., Luna C.M., Oberschelp G.P.J., Harrand L., Cabello M.N. (2020). Interactions between mineral fertilization and arbuscular mycorrhizal fungi improve nursery growth and drought tolerance of *Prosopis alba* seedlings. Agroforest Syst..

[60] Salcido S., Prieto J.L., Santana E., Chávez J. (2021). Pinus greggii engelm.: Respuesta a la inoculación micorrícica controlada y a la fertilización en vivero. Agrociencia.

[61] Klironomos J.N. (2003). Variation in plant response to native and exotic arbuscular mycorrhizal fungi. Ecology.

[62] Bustos F., González M., Donoso P., Gerding V., Donoso C., Escobar B. (2008). Efectos de distintas dosis de fertilizante de liberación controlada (Osmocote®) en el desarrollo de plantas de coigüe, raulí y ulmo. Bosque.

[63] Dexheimer J., Pargney J.C. (1991). Comparative anatomy of the host-fungus interface in mycorrhizas. Experientia.

[64] Ditengou F., Lapeyrie F. (2000). Hypaphorine from the ectomycorrhizal fungus *Pisolithus tinctorius* counteracts activities of indole-3-acetic acid and ethylene but not synthetic auxins in eucalypt seedlings. Mol. Plant-Microbe Interact..

[65] Horan D., Chilvers G., Lapeyrie F. (1988). Time sequence of the infection process in eucalypt ectomycorrhizas. New Phytol..

[66] Felten J., Kohler A., Morin E., Bhalerao R., Palme K., Martin F.S., Ditengou F., Legué V. (2009). The ectomycorrhizal fungus *Laccaria bicolor* stimulates lateral root formation in poplar and Arabidopsis through auxin transport and signaling. Plant Physiol..

[67] Sukumar P., Legué V., Vayssières A., Martin F., Tuskan G.A., Kalluri U.C. (2013). Involvement of auxin pathways in modulating root architecture during beneficial plant-microorganism interactions. Plant Cell Environ..

[68] Vayssières A., Pěnčík A., Felten J., Kohler A., Ljung K., Martin F., Legué V. (2015). Development of the *Poplar-Laccaria bicolor* Ectomycorrhiza Modifies Root Auxin Metabolism, Signaling, and Response. Plant Physiol..

[69] Boyle C., Robertson W., Salonius P. (1987). Use of slurries of mycorrhizal fungi as inoculum for commercial tree seedling nurseries. Can. J. For. Res..

[70] Carrillo C. (2000). Técnicas de Micorrización En Vivero Con Hongos Ectomicorrícicos. Experiencias Realizadas en el Centro Nacional de Mejora Forestal “El Serranillo”.

[71] Castellano M.A., Trappe J.M., Molina R. (1985). Inoculation of container-grown Douglas-fir seedlings with basidiospores of *Rhizopogon vinicolor* and *R. colossus*: Effects of fertility and spore application rate. Can. J. For. Res..

[72] Khasa P.D., Sigler L., Chakravarty P., Dancik B.P., Erickson L., Mc Curdy D. (2001). Effect of fertilization on growth and ectomycorrhizal development of container-grown and bare-root nursery conifer seedlings. New For..

[73] Hilszcanska D., Sierota Z. (2006). Persistence of ectomycorrhizas by *Thelephora terrestres* on outplanted *Scots pine* seedlings. Acta Mycol..

[74] Dixon R.K., Behrns G.T., Garrett H.E., Cox G.S., Sander I.L. (1985). Synthesis of Ectomycorrhizae on Container-Grown Oak Seedlings. South. J. Appl. For..

[75] Landis T.D., Landis T.D., Tinus R.W., McDonald S.E., Barnett J.P. (1990). Containers: Types and functions. Agric. Handbk. 674. The Containers Tree Nursery Manual, Volumen 2.

[76] Honrubia M., Torres P., Díaz G., Cano A. (1992). Manual Para Micorrizar Plantas En Viveros Forestales.

[77] Davies F.T., Svenson S.E., Cole J.C., Phavaphutanon L., Duray S.A., Olalde-Portugal V., Meier C.E., Bo S.H. (1996). Non-nutritional stress acclimation of mycorrhizal woody plants exposed to drought. Tree Physiol..

[78] Singer R. (1969). Mycoflora Australis.

[79] Torres-Díaz C., Valladares M.A., Acuña-Rodríguez I.S., Ballesteros G.I., Barrera A., Atala C., Molina-Montenegro M.A. (2021). Symbiotic Interaction Enhances the Recovery of Endangered Tree Species in the Fragmented Maulino Forest. Front. Plant Sci..

[80] Zúñiga R., Alberdi M., Reyes-Díaz M., Olivares E., Hess S., Bravo L.A., Corcuera L.J. (2006). Seasonal changes in the photosynthetic performance of two evergreen *Nothofagus* species in south central Chile. Rev. Chil. De Hist. Nat..

[81] Sebastiana M., da Silva A.B., Matos A.R., Alcântara A., Silvestre S., Malhó R. (2018). Ectomycorrhizal inoculation with Pisolithus tinctorius reduces stress induced by drought in cork oak. Mycorrhiza.

[82] Morte A., Dieste C., Díaz G., Gutiérrez A., Navarro A., Honrubia M. Production of *Terfezia olbiensis* mycelial inoculum in a bioreactor. Proceedings of the 1st Symp Champignons Hypoges du Basin Mediterraneen.

[83] Ortega U., Duñabeitia M., Menendez S., Gonzalez-Murua C., Majada J. (2004). Effectiveness of mycorrhizal inoculation in the nursery on growth and water relations of *Pinus radiata* in different water regimes. Tree Physiol..

[84] Baltruschat H., Fodor J., Harrach B.D., Niemczyk E., Barna B., Gullner G., Janeczko A., Kogel K.H., Schäfer P., Schwarczinger I. (2008). Salt tolerance of barley induced by the root endophyte Piriformospora indica is associated with a strong increase in antioxidants. New Phytol..

[85] Cartes Rodríguez E., Acevedo Tapia M., González Ortega M., Álvarez C., García Rivas E., Mena Marín P.P. (2019). Manual de Manejo de Riego y Fertilización en Viveros de Plantas a Raíz Cubierta.

[86] Landis T.D., Landis T.D., Tinus R.W., McDonald S.E., Barnett J.P. (1989). Mineral nutrients and fertilization. The Container Tree Nursery Manual, Volume 4. Agric. Handbk. 674.

[87] Arenas F., Navarro-Ródenas A., Chávez D., Gutiérrez A., Pérez-Gilabert M., Morte A. (2018). Mycelium of Terfezia claveryi as inoculum source to produce desert truffle mycorrhizal plants. Mycorrhiza.

[88] Iverson R.D., Duryea M.L., Landis T.D., Perry C.R. (1984). Planting-Stock Selection: Meeting Biological Needs and Operational Realities. Forestry Nursery Manual: Production of Bareroot Seedlings.

