# Supplementary material for: Early Effects of Mycorrhizal Fungal Inoculum and Fertilizer on Morphological and Physiological Variables of Nursery-Grown Nothofagus alessandrii Plants
Source: Plants (Basel). 2023 Mar 31;12(7):1521. doi: 10.3390/plants12071521 (PMC10096678; doi:10.3390/plants12071521)
Supplement: Supplementary file 1 [file plants-12-01521-s001.zip › plants-2184512-supplementary.pdf]

Supplementary Materials

Table S1. Significance level for the main effects (TP = Plant type, F = Fertilization, M = mycorrhization) and their interactions in the morphological attributes height and diameter of *Nothofagus alessandrii*.

Factor	Significance level (*** p < 0.001, ** p < 0.01, * p <0.05)	
	Height	Diameter
TP	***	***
F	n.s.	n.s.
TP*F	n.s.	n.s.
M	n.s.	***
TP*M	n.s.	***
F*M	n.s.	n.s.
TP*F*M	n.s.	n.s.

Table S2. Significance level for the main effects (TP = Plant type, F = Fertilization, M = mycorrhization) and their interactions in the morphological attributes Stem:Root Index (SRI) and Slenderness Index (SI) of *Nothofagus alessandrii* seedlings.

Factor	Significance level (*** p < 0.001, ** p < 0.01, * p <0.05, · p <0.1)	
	SRI	SI
TP	***	***
F	***	n.s.
TP*F	n.s.	*
M	n.s.	n.s.
TP*M	n.s.	*
F*M	.	**
TP*F*M	*	n.s.

Table S3. Macro and micro nutrients concentration in leaves of *Nothofagus alessandrii* in one and two-season plants (P1 and P2) (TP = Plant type, F = Fertilization, M = mycorrhization)

TP	F	M	N Dumas (%)	N Kjeldahl (%)	P (%)	K (%)	Ca (%)	Mg (%)	Mn (ppm)	Zn (ppm)	Cu (ppm)	Fe (ppm)	B (ppm)
P1	F1	M0	2.34	2.17	0.46	0.69	0.39	0.55	946	61	6	606	89
P1	F1	M2	2.43	2.26	0.42	0.7	0.32	0.45	772	61	6	257	83
P1	F1	M1	2.44	2.26	0.46	0.63	0.34	0.46	569	55	10	245	83
P1	F1	M1	2.22	2.06	0.39	0.58	0.36	0.5	738	60	5	258	90
P1	F2	M0	2.72	2.52	0.69	0.74	0.36	0.45	1186	132	15	280	198
P1	F2	M0	2.61	2.42	0.68	0.74	0.35	0.47	1291	128	10	266	228
P1	F1	M0	2.44	2.26	0.39	0.61	0.38	0.5	977	63	4	242	82
P1	F2	M1	2.79	2.59	0.78	0.79	0.3	0.44	1387	110	10	188	197
P1	F2	M2	2.76	2.56	0.73	0.87	0.32	0.41	1444	100	11	160	187
P1	F2	M1	2.81	2.6	0.75	0.82	0.33	0.43	1950	117	12	235	196
P1	F1	M0	2.48	2.3	0.56	0.62	0.35	0.45	799	57	7	177	88
P1	F2	M2	2.8	2.59	0.75	0.91	0.38	0.46	1792	111	21	201	190
P1	F2	M0	2.67	2.48	0.72	0.73	0.37	0.46	1687	103	13	197	187
P1	F1	M1	2.36	2.19	0.54	0.76	0.4	0.46	1015	64	7	241	101
P1	F2	M2	2.58	2.39	0.69	0.8	0.35	0.44	1408	105	12	210	209
P1	F2	M1	2.64	2.44	0.67	0.84	0.33	0.49	829	103	10	216	223
P1	F1	M2	2.47	2.29	0.56	0.71	0.37	0.45	1055	76	10	232	86
P1	F1	M2	2.12	1.96	0.5	0.65	0.37	0.53	1057	64	8	349	91
P2	F2	M2	2.48	2.3	0.79	1.03	0.42	0.45	1071	97	12	203	203
P2	F1	M2	1.9	1.76	0.46	0.56	0.39	0.45	621	45	5	216	94
P2	F1	M2	1.97	1.83	0.51	0.61	0.4	0.44	830	55	6	217	98
P2	F1	M1	1.95	1.8	0.46	0.67	0.45	0.43	841	51	5	227	103
P2	F1	M0	2.05	1.9	0.6	0.65	0.51	0.51	1007	59	12	211	108
P2	F1	M1	1.86	1.73	0.45	0.73	0.38	0.44	613	50	6	175	106
P2	F1	M2	1.87	1.73	0.5	0.59	0.4	0.46	1005	47	7	182	109
P2	F1	M0	2.19	2.03	0.52	0.78	0.46	0.46	631	56	8	189	115
P2	F2	M0	2.6	2.41	0.82	1.06	0.4	0.47	1088	88	12	201	235
P2	F2	M1	2.35	2.18	0.8	0.91	0.35	0.43	813	88	13	145	221
P2	F1	M1	1.86	1.73	0.51	0.75	0.43	0.48	784	57	7	276	109
P2	F2	M0	2.59	2.4	0.61	0.8	0.39	0.41	655	92	11	182	167
P2	F2	M2	2.35	2.18	0.75	0.9	0.45	0.46	1002	90	12	287	199
P2	F2	M0	2.48	2.3	0.98	1.22	0.51	0.54	1081	105	9	214	258
P2	F2	M1	2.36	2.19	0.74	0.92	0.45	0.45	1024	81	11	304	228
P2	F2	M2	2.28	2.12	0.73	1.05	0.43	0.47	760	84	9	263	243
P2	F2	M1	2.27	2.11	0.82	1.03	0.39	0.5	824	95	9	355	265

Table S4. The significance level for the main effects (TP = Plant type, F = Fertilization, M = mycorrhization) and their interactions on the concentration of nutrients in the leaves of *Nothofagus alessandrii* in one– and two–season plants (P1 and P2).

Factor	Significance level (***p < 0.001, **p < 0.01, *p <0.05, ·p <0.1)										
	**N Dumas**	**N Kjeldahl**	**P**	**K**	**Ca**	**Mg**	**Mn**	**Zn**	**Cu**	**Fe**	**B**
F	***	***	***	***	n.s.	n.s.	***	***	***	n.s.	***
M	*	*	n.s.	n.s.	.	.	n.s.	n.s.	n.s.	n.s.	n.s.
TP	***	***	.	**	***	n.s.	**	***	n.s.	n.s.	**
FxM	n.s.	n.s.	n.s.	n.s.	n.s.	n.s.	n.s.	n.s.	n.s.	n.s.	n.s.
FxTP	n.s.	n.s.	n.s.	**	n.s.	n.s.	*	*	n.s.	.	n.s.
MxTP	*	*	n.s.	.	n.s.	n.s.	n.s.	n.s.	n.s.	n.s.	n.s.
FXMXTP	n.s.	n.s.	n.s.	n.s.	n.s.	n.s.	n.s.	n.s.	n.s.	n.s.	n.s.

Table S5. Level of significance for main effects (TP = Plant type, F = Fertilization, M = mycorrhization) and their interactions on photosynthesis, stomatal conductance, and intracellular CO_2 of *Nothofagus alessandrii* plants.

Factor	Significance level (***p < 0.001, **p < 0.01, *p <0.05)		
	**Photosynthesis**	**Stomatal conductance**	**Intracellular carbon**
TP	n.s.	***	***
F	**	n.s.	**
TP*F	n.s.	*	n.s.
M	n.s.	*	n.s.
TP*M	**	n.s.	***
F*M	n.s.	n.s.	n.s.
TP*F*M	n.s.	n.s.	n.s.
